# A Perspective on *Hypericum perforatum* Genetic Transformation

**DOI:** 10.3389/fpls.2016.00879

**Published:** 2016-06-24

**Authors:** Weina Hou, Preeti Shakya, Gregory Franklin

**Affiliations:** ^1^Centre for the Research and Technology of Agro-Environment and Biological Sciences, University of MinhoBraga, Portugal; ^2^Department of Integrative Plant Biology, Institute of Plant Genetics of the Polish Academy of SciencesPoznan, Poland

**Keywords:** *Agrobacterium tumefaciens*, *A. rhizogenes*, *Hypericum perforatum*, hairy root culture, biolistic bombardment, metabolic engineering, regeneration

## Abstract

*Hypericum perforatum* (St John's wort) is a reservoir of diverse classes of biologically active and high value secondary metabolites, which captured the interest of both researchers and the pharmaceutical industry alike. Several studies and clinical trials have shown that *H. perforatum* extracts possess an astounding array of pharmacological properties. These properties include antidepressant, anti-inflammatory, antiviral, anti-cancer, and antibacterial activities; and are largely attributed to the naphtodianthrones and xanthones found in the genus. Hence, improving their production via genetic manipulation is an important strategy. In spite of the presence of contemporary genome editing tools, genetic improvement of this genus remains challenging without robust transformation methods in place. In the recent past, we found that *H. perforatum* remains recalcitrant to *Agrobacterium tumefaciens* mediated transformation partly due to the induction of plant defense responses coming into play. However, *H. perforatum* transformation is possible via a non-biological method, biolistic bombardment. Some research groups have observed the induction of hairy roots in *H. perforatum* after *Agrobacterium rhizogenes* co-cultivation. In this review, we aim at updating the available methods for regeneration and transformation of *H. perforatum*. In addition, we also propose a brief perspective on certain novel strategies to improve transformation efficiency in order to meet the demands of the pharmaceutical industry *via* metabolic engineering.

## Introduction

*Hypericum perforatum* is one of the most important and well-known species of the *Hypericum* genus, which has been appreciated by Greek herbalists for its medicinal value since the first century A.D. Several studies and clinical trials have shown that *H. perforatum* extracts possess an astounding array of pharmacological properties. The clinical efficacies of *H. perforatum* extracts in the therapy of mild to moderate depression have been confirmed in many studies (Lecrubier et al., 2002; Butterweck, 2003). Many other important pharmaceutical properties of *H. perforatum* including antiviral (Schinazi et al., 1990), anticancer (Agostinis et al., 2002), neuroprotective (Silva et al., 2004), antioxidant (Silva et al., 2005), and wound healing (Yadollah-Damavandi et al., 2015) activities have also been reported. Since treating humans and animals with *H. perforatum* extracts does not result in any serious adverse side effects (Trautmann-Sponsel and Dienel, 2004), use of this medicinal herb has increased dramatically during the past decade. Because of its well-established market position, popularity, and efficacy, *H. perforatum* is reputed as one of the best-selling herbs today. *H. perforatum* products are currently sold as dietary supplements, anti-depressive agents, relaxants, and mood enhancers in many countries.

*H. perforatum* cell and tissue cultures have been attempted with the main focus being to produce pharmaceutically important compounds under controlled conditions. However, large-scale production of secondary metabolites could not be achieved so far using *in vitro* cultures due to low performance and unreliable yield of the products. Although, significant improvements in product yields have been achieved through conventional biochemical approaches combined with the manipulation of culture process, the results are not reproducible. Plant metabolic pathway engineering would allow us to improve the production of major compounds in *H. perforatum* by overexpressing specific genes. However, metabolic engineering of this genus has so far not been attempted due to the lack of an efficient transformation method.

Plant transformation is an indispensable tool for crop improvement, plant functional genomics, genome editing, synthetic biology, etc. (Sainsbury and Lomonossoff, 2014; Xu et al., 2014; Hwang et al., 2015; Nester, 2015). Success of transformation in non-model plants is generally based on two important principles: (1) foreign genes could be introduced into a plant cell through various methods and its genetic makeup could be altered and (2) plant cells are totipotent, which means in principle that every cell contains all the genetic information necessary to regenerate into a complete plant under optimal conditions. Therefore, the efficiency of gene delivery into target cells and the ability to recover plants from those transformed cells are the two major factors critically contributing to the recovery of transgenic plants. In spite of the availability of excellent regeneration methods *via* organogenesis and somatic embryogenesis in *H. perforatum*, the recovery of transgenic plants remains challenging. Although *Agrobacterium rhizogenes* and biolistics mediated transformation of *H. perforatum* has been reported, these protocols could not meet the vast needs of functional genomic research. *Agrobacterium tumefaciens* mediated transformation is the most preferred method of gene transfer due to frequent single copy transgene integration into the plant genome and low incidence of transgene silencing. The advantages of simplicity, affordable costs, lower transgenic rearrangement, ability for long DNA segment transfer, and preferential integration of foreign genes into transcriptionally active regions make *A. tumefaciens*-mediated transformation an attractive method (Kumar et al., 2013). Although this method could be useful for metabolic engineering and functional genomic studies in *H. perforatum*, plant recalcitrance against *A. tumefaciens* mediated transformation is a major concern. In this article, we discuss the present status and future perspectives of genetic transformation of *H. perforatum*.

## Cellular totipotency of *H. perforatum*

Cellular totipotency of *H. perforatum* has been demonstrated in several reports. Originally, *in vitro* regeneration of *H. perforatum* has been investigated as an option for multiplication of elite plants and production of valuable phytopharmaceuticals. In particular, the effect of plant growth regulator (PGR) combinations on secondary metabolite concentration has been intensively studied in cell and tissue culture. As a result, several methods of plant regeneration and micropropagation are available today.

Basically, *in vitro* plant regeneration of *H. perforatum* is relatively simple and quick. *In vitro* regeneration of *H. perforatum* has been achieved from several types of explants (Table 1), including whole seedlings (Cellarova et al., 1992), leaves (Pretto and Santarem, 2000; Pasqua et al., 2003; Franklin and Dias, 2006), nodal segments (Santarém and Astarita, 2003), root segments (Zobayed and Saxena, 2003; Franklin and Dias, 2006), hypocotyls (Murch et al., 2000; Franklin and Dias, 2006), stems (Zobayed and Saxena, 2003), shoot tips (Zobayed and Saxena, 2003), organogenic nodules derived from cell suspension culture (Franklin et al., 2007), and thin cell layers (Franklin and Dias, 2011). Root explants responded better than the shoot tip, leaf, hypocotyl, or stem explants in terms of thidiazuron-induced shoot organogenesis, whereas, the lowest number of regenerants was found in shoot tip explants (Zobayed and Saxena, 2003). Plants could be produced on medium augmented with various PGR combinations. Although the general requirement for shoot regeneration is a high cytokinin/auxin ratio in most species, *H. perforatum* showed efficient direct shoot regeneration on a low cytokinin/auxin ratio (Pasqua et al., 2003; Franklin and Dias, 2006). On the other hand, for callus mediated indirect shoot regeneration, *H. perforatum* needs a high cytokinin/auxin ratio. Interestingly, plants could be efficiently regenerated from root explants on basal medium (Franklin and Dias, 2006) and on medium supplemented with IAA (Goel et al., 2008).

**Table 1:** *****In vitro*** plant regeneration of ***H. perforatum*****.

**Explant**	**PGRs tested**	**Major result**	**References**
Seedling	BA	Plant regeneration	Cellarova et al., 1992
Halved leaves	2,4-D, BA, KIN, IBA	Callus initiation and shoot organogenesis	Pretto and Santarem, 2000
Isolated anther	NAA, BA	Plant regeneration from isolated anthers	Murch and Saxena, 2002
Shoot tip, hypocotyl, root, and whole seedling	Thidiazuron, NAA, IBA, IAA	Best regeneration potential of root explants	Zobayed and Saxena, 2003
Leaf discs and stem segments	2,4-D, KIN	Leaf disks are better than stem segments for shoot regeneration	Ayan et al., 2005
Root, hypocotyl, and leaves from *in vitro* grown seedlings	BA, IAA	Organogenesis and embryogenesis in several genotypes	Franklin and Dias, 2006
Organogenic nodules obtained from cell suspension culture	BA, NAA	Plant regeneration	Franklin et al., 2007
*In vitro* grown roots	IAA, IBA, NAA, KIN	Established liquid culture medium most suitable for culturing roots	Goel et al., 2008
Nodal segments from *in vitro* gown shoots	BA	Used different liquid cultures, semisolid, partial immersion, paper bridge, and total immersion for shoot organogenesis	Savio et al., 2011
Petals	IAA, IBA, KIN	Shoot regeneration from petals dependent on age of buds	Palmer and Keller, 2011
Thin cell layers of organogenic nodules	BA, NAA	Regulation of shoot, root and root hair development by chlorogenic acid	Franklin and Dias, 2011

Most of the regeneration studies are restricted to a single genotype (Murch et al., 2000; Pretto and Santarem, 2000; Zobayed and Saxena, 2003; Zobayed et al., 2004). We have established a genotype-independent plant regeneration protocol and elucidated the specific pathway of plant regeneration in *H. perforatum* (Franklin and Dias, 2006). There was no significant difference in the percentage of regeneration and number of shoots/explants between the tested genotypes indicating regeneration in *H. perforatum* is genotype independent. On the other hand, the explant type (hypocotyl, leaf, or root) had a significant effect on the regeneration of shoots (Franklin and Dias, 2006). Similar variation in the regeneration frequency of shoots based on explant types on the same thidiazuron concentration was also reported previously in *H. perforatum* (Zobayed and Saxena, 2003). Hence, from the results reported in the literature, *H. perforatum* regeneration response is clearly a PGR-driven explant-dependent phenomenon.

Age of the explant source also affected the regeneration potential of leaf, hypocotyl, and petal explants (Franklin and Dias, 2006; Goel et al., 2008). In contrast, age did not affect the morphogenetic potential of root segment explants (Franklin and Dias, 2006). Age-independent regeneration of root segments might be due to the high metabolic activity and faster cell division of roots due to continuous meristematic activity nearer to the root tip. Orientation of leaf explants on the medium also had a distinct effect on regeneration. While leaves with their adaxial side touching the medium exhibited high frequencies of regeneration, leaves with the opposite surface contacting the medium failed to show any response.

**Figure 1:**
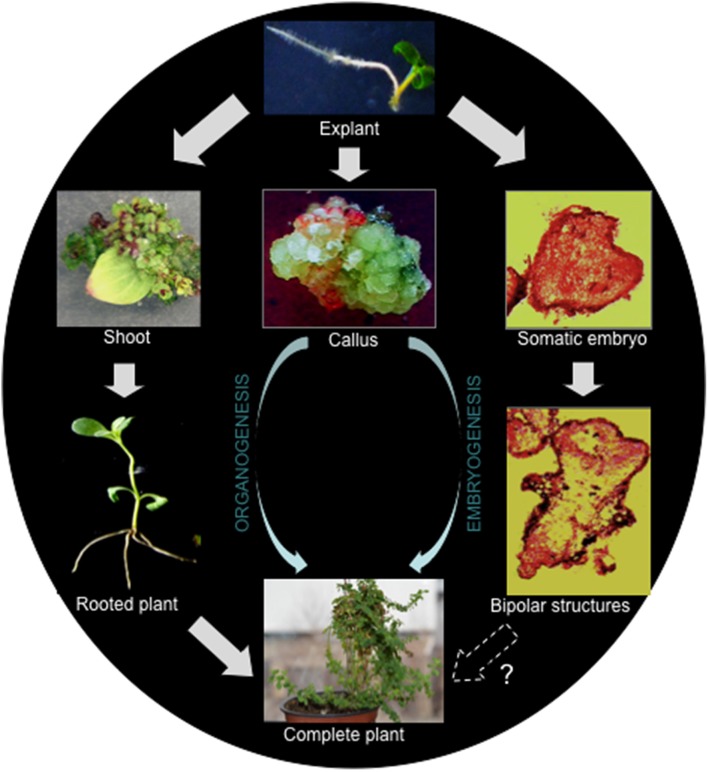
**Regeneration pathways leading to the regeneration of ***H. perforatum*** as revealed from our previous report (Franklin and Dias, 2006)**.

Generally, there are two important pathways leading to regeneration of a new plant from cultured explants, organogenesis, and somatic embryogenesis (Figure 1). A process in which an organ (e.g., shoot or root) is initiated and developed is known as organogenesis. On the other hand, the process of formation of an embryo, which is developed from somatic cells, is called somatic embryogenesis. While the emergence of a unipolar primordium or a bipolar embryo are the typical characteristics of organogenesis and somatic embryogenesis, respectively. During the above processes, if de-differentiation (callus formation) is involved, they are termed indirect regeneration.

In *H. perforatum* regeneration has been demonstrated *via* both embryogenesis and organogenesis in the same culture (Franklin and Dias, 2006). In this study, meristematic cells formed from the sub-epidermal layer developed into two functionally different globular structures simultaneously. The globular structures, which were attached to the explant developed into shoots, while the others detached from the explant underwent embryogenesis. Embryogenesis progressed from the globular embryos to the cotyledon stage *via* heart-shaped and torpedo-stage embryos. Cotyledonary embryos did not develop into plants as they failed to establish root systems. It should be noted that indirect regeneration is better suited for generating transgenic plants than direct regeneration, as the selection of transgenic callus is usually straightforward and allows efficient enrichment of transformed tissue before regeneration.

## DNA delivery into *H. perforatum* plant cells

### Agrobacterium mediated transformation

*A. tumefaciens*-mediated transformation is the most efficient and commonly used technique in plant genetic engineering. On the other hand, hairy root cultures established by *A. rhizogenes*-mediated transformation often sustain stable productivity in hormone-free culture conditions resulting in large amounts of secondary metabolites accumulating (Oksman-Caldentey and Sévon, 2002).

*Agrobacterium* is called the “natural genetic engineer” because of its natural capacity to infect plants and introduce a piece of DNA (T-DNA) from its tumor inducing (Ti) or root inducing (Ri) plasmid into plant cells *via* a process known as “T-DNA transfer.” Once inside the plant cell, the T-DNA (transferred DNA) is transported into the nucleus where it stably integrates into the plant genome. T-DNA encodes genes for the synthesis of auxin, cytokinin, and opine. Hence, T-DNA integration into the host genome results in an imbalance of host cell auxin–cytokinin ratios, which leads to uncontrolled cell division and the development of crown galls or hairy roots and opine synthesis. Opines are used as the main food resource by *Agrobacterium*. With neither the T-DNA able to be transcribed in *Agrobacterium* nor opines metabolized by plants the T-DNA transfer process is a molecular niche for *Agrobacterium*'s survival. This natural process is evidenced in several plant species (e.g., rose, grape, stone fruit, pome, tomato) and considered as a disease. This disease causing T-DNA transfer process has been exploited as a tool to introduce genes into plants. Today, *A. tumefaciens*-mediated transformation is the preferred method for functional genomics because of its simplicity and frequent single copy transgene integration into the host genome.

Although *A. tumefaciens* mediated *H. perforatum* transformation has not yet been reported, induction of hairy roots after co-cultivation with *A. rhizogenes* has been reported (Table 2). Although strains ATCC 15834 and A4 strains could produce hairy roots, *A. rhizogenes* strain K599 did not induce hairy root formation (Santarem et al., 2008). On the other hand, *A. rhizogenes* strain like A4, LBA9402, could not induce hairy roots in *H. perforatum* cv. Helos (Franklin et al., 2007). The seemingly contradictory results between groups clearly emphasize the complexity of *A. rhizogenes* mediated transformation of *H. perforatum*.

**Table 2:** *****A. rhizogenes*** mediated transformation of ***H. perforatum*****.

***A. rhizogenes* strain**	**Explant**	**Molecular confirmation**	**References**
ATCC 15834	Root and leaf	PCR and southern blot analysis of rolC gene	Di Guardo et al., 2003
A4	Epicotyls	PCR amplification of GUS gene	Vinterhalter et al., 2006
LBA9402 and A4	Root, leaf, epicotyl, and organogenic nodules	No hairy root induction	Franklin et al., 2007
ATCC 15834	Leaf and root fragments	PCR amplification of rolC gene	Bertoli et al., 2008
K599	Adventitious shoots	No hairy root induction	Santarem et al., 2008, 2010
A4	Root segments	PCR amplification of rolB and rolC genes	Tusevski et al., 2013b, 2014

Hairy root cultures could be established from *H. perforatum* epicotyls co-cultivated with *A. rhizogenes* strain A4 containing GUS (β-glucuronidase) gene inserted into the Ri plasmid pRiA4 (Vinterhalter et al., 2006). These hairy roots exhibited high potential for spontaneous regeneration into whole transgenic plants. The presence of GUS gene in the hairy root and shoot cultures was determined by PCR analysis. Recently, this group studied the effect of sucrose concentration on shoot regeneration potential of *H. perforatum* hairy roots clones obtained from their previous study (Vinterhalter et al., 2006) and found that up to 2% sucrose promoted intense shoot regeneration (Vinterhalter et al., 2015).

Co-cultivation of root segments with *A. rhizogenes* strain A4 resulted in hairy root production of *H. perforatum* (Tusevski et al., 2013b, 2014). Transgenic nature of the hairy root cultures was demonstrated by PCR amplification of rolB gene in DNA isolated from the roots. These authors have also found several important secondary metabolites (phenolic acids, flavonol glycosides, flavonoid aglycones, flavan-3-ols, and xanthones) in hairy roots of *H. perforatum* (Tusevski et al., 2013b, 2014). This group has also compared the production of phenolic compounds between dark-grown hairy root cultures and those grown with a 16 h photoperiod, which revealed marked differences in phenolic acids, flavonols, flavan-3-ols, and xanthones between those cultures (Tusevski et al., 2013a). Similarly, hairy root clones with elevated levels of hyperoside, chlorogenic acid, and hypericin were obtained from leaf and root fragments co-cultivated with *A. rhizogenes* strain ATCC 15834 (Bertoli et al., 2008). Futhermore, hypericin was found at elevated levels in adventitious shoots of *H. perforatum* after co-cultivation with *A. rhizogenes* strain K599, despite the co-cultivation not resulting in hairy root formation (Santarem et al., 2008, 2010). Similarly, co-cultivation with *A. tumefaciens* and *A. rhizogenes* enhanced secondary metabolite production in *H. perforatum* cell suspension culture (Tusevski et al., 2015).

### *H. perforatum* recalcitrance to *Agrobacterium* infection

Neither *A. rhizogenes* (LBA99402 and A4) nor *A. tumefaciens* (LBA4404 and EHA105) could infect *H. perforatum* tissues in our studies. Various explants (leaf blade, petiole, stem, and root segments) were co-cultivated with *A. tumefaciens* and *A. rhizogenes* carrying a binary vector pCAMBIA1301 which carries the HPT (hygromycin phosphotransferase) gene as the selection marker and GUS interrupted with a eukaryotic intron (GUS-INT) as the reporter gene. The presence of an intron in the GUS gene permits gene expression only in eukaryotic cells such as plant cells. When assayed for transient GUS gene expression, none of the explants showed blue foci (Franklin et al., 2007). This was irrespective of vir gene induction or addition of an antioxidant (butylated hydroxytoluene, BHT), thiol compounds (cysteine), or ethylene inhibitors (AgNO_3_ and aminoethoxyvinylglycine) to the co-cultivation medium. We presumed that antimicrobial secondary metabolites might be the reason for the inability of *Agrobacterium* to infect these explants.

In order to avoid antimicrobial compounds such as hypericins in the explants, we used organogenic nodule explants derived from cell suspension culture that lack hypericin glands (Franklin et al., 2007). Upon co-cultivation with *A. tumefaciens* or *A. rhizogenes*, these explants started to become brown within one day and subsequently become necrotic within 10 days. They did not show any transient GUS expression or callus formation when grown on selection medium containing antibiotic. On the other hand, under non-selective conditions, all the explants co-cultivated with *A. tumefaciens* and *A. rhizogenes* regained their normal growth within 5 days and produced calluses comparable to the control explants. In spite of the browning occurring after *Agrobacterium* co-cultivation, genomic DNA isolated from the explants did not show any fragmentation indicating that the incompatibility of *Agrobacterium*-mediated transformation in *H. perforatum* is not due to necrosis induced by programmed cell death as reported in maize (Hansen, 2000).

When the co-cultivation medium was augmented with BHT, two explants co-cultivated with *A. tumefaciens* strain EHA105 and one explant co-cultivated with *A. tumefaciens* strain LBA4404 showed blue foci in the GUS assay. Whereas, explants co-cultivated in the presence of other antioxidants and ethylene inhibitors as well as the shoots obtained from the calluses maintained in non-selective medium after co-cultivation did not show GUS gene expression. Even though the calluses obtained under non-selective conditions regenerated shoots as the control, none of them were transgenic. A number of plant species previously considered recalcitrant to *A. tumefaciens* became transformable upon supplementing antioxidants (Das et al., 2002; Frame et al., 2002) and ethylene inhibitors (Han et al., 2005; Petri et al., 2005; Seong et al., 2005) in the co-cultivation medium. This is mainly because of the fact that these scavengers could suppress the oxidative burst or ethylene production during plant–*Agrobacterium* interactions. However, in our case the tested antioxidants and ethylene inhibitors added to the co-cultivation medium neither prevented tissue browning nor favored transformation.

### *H. perforatum* plant defense response against *Agrobacterium*

The mechanism of *H. perforatum* recalcitrance against *Agrobacterium* infection was studied using cell suspension cultures (Franklin et al., 2008, 2009a). Briefly, *H. perforatum* cell suspension culture was challenged with *A. tumefaciens* strain EHA105 and *A. rhizogenes* strain A4 both containing plasmid pCAMBIA1301. After different post inoculation periods (0, 6, 12, and 24 h), both the plant cells and bacteria were analyzed. A typical biphasic ROS (reactive oxygen species) burst followed by darkening of *H. perforatum* cells was observed. In spite of ROS production *H. perforatum* cells did not undergo an obvious apoptotic process, while both *A. tumefaciens* and *A. rhizogenes* reached 99% mortality within 12 h of co-cultivation (Franklin et al., 2008). On the other hand, *A. tumefaciens* co-cultivation with tobacco BY2 cells under the same conditions lead to successful T-DNA transfer.

In addition to ROS production, genes encoding important enzymes of the phenylpropanoid pathway such as phenylalanine ammonia lyase (PAL), 4-coumarate:CoA ligase (4CL), and benzophenone synthase (BPS) were upregulated which would eventually lead to alteration of the profile of secondary metabolites. Analysis of the soluble phenolic fraction revealed an enormous increase in xanthone concentration and the emergence of many xanthones in *H. perforatum* cells after *Agrobacterium* co-cultivation was observed, while flavonoid content remained unaffected (Franklin et al., 2009a). Recently, we studied changes in *H. perforatum* cell wall fractions and cell wall bound phenolic compounds in response to *A. tumefaciens* elicitation (Singh et al., 2014). This study revealed that lignin content was significantly increased in *H. perforatum* cell walls after *A. tumefaciens* elicitation (0.085–0.24 mg/mg dry weight cell wall) implying that *H. perforatum* reinforced its cell wall as a protective measure against *A. tumefaciens* infection. Similarly, flavonoid (e.g., quercetin, quercetrin etc.) content was also significantly higher in the cell walls of elicited cells compared to controls. Hence, in addition to PAL, 4CL, and BPS (Franklin et al., 2009a), chalcone synthase (CHS) is also upregulated after elicitation (Singh et al., 2014). While those xanthones produced in response to *A. tumefaciens* elicitation were incorporated into the soluble phenolic fraction, flavonoids were actually incorporated into the cell wall. This swift change in the secondary metabolites increased the cellular antioxidant and antimicrobial competence compared to the control cells revealing that this change plays a dual role in the plant cells; as antioxidants to protect the cells from oxidative damage and as phytoalexins to impair the pathogen growth upon *Agrobacterium* interaction.

**Figure 2:**
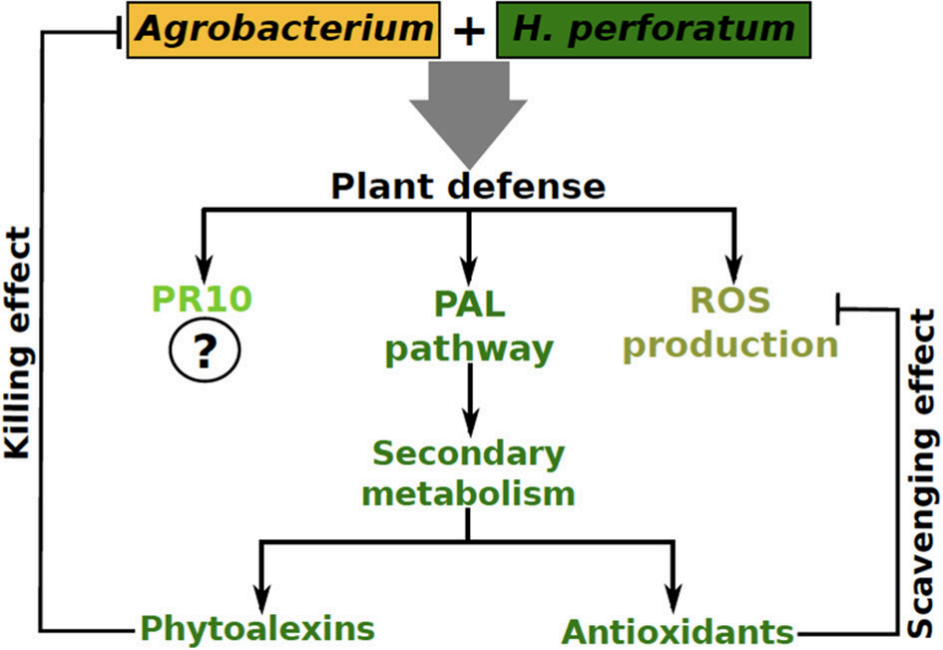
**A model summarizing plant defense activation in ***H. perforatum*** upon its interaction with ***Agrobacterium*****.

Thus, we provided the first evidence for a typical oxidative burst combined with the upregulation of phenylpropanoid pathway genes in response to *Agrobacterium* co-cultivation, which could prevent T-DNA transfer. Recently, upregulation of a pathogenesis related 10 (PR10) gene (Sliwiak et al., 2015) in *H. perforatum* upon *A. tumefaciens* co-cultivation has been reported (Kosuth et al., 2013). Based on the above observations, we believe that recalcitrant plants could mobilize their antioxidant, antimicrobial and PR defense machinery against *Agrobacterium* (Figure 2).

Considering all the studies conducted so far in our laboratory and by others, the emerging depiction is that in both compatible and incompatible plant-*Agrobacterium* interactions, an initial defense response is induced. In the case of compatible interactions, despite the initial transient activation of basal host defense, the subsequent transfer of virulence factors might lead to the suppression of plant defense, resulting in successful transformation as observed in tobacco (Veena et al., 2003; Franklin et al., 2008). By contrast, in incompatible interactions the initially evoked plant defense response is long lasting (and successful), therefore, affecting the bacterium and preventing T-DNA transfer into plant cells, as observed in *H. perforatum* (Franklin et al., 2008), making these plants recalcitrant to *Agrobacterium*-mediated transformation.

### Biolistic-mediated transformation of *H. perforatum*

Biolistic technology (particle bombardment) is a useful technique used in the genetic manipulation of many crop improvement programs. In this method, the vector carrying the gene of interest is coated on metal particles and bombarded on target tissues with a high force/pressure by a biolistic device or gene gun (Kikkert et al., 2005). The biolistic method not only allows the expression of multiple transgenes in the target tissue, which can be achieved by fusion of genes within the same plasmid that is then bombarded into the target tissues, but also serves as an alternative method to achieve transient or stable transformation in *Agrobacterium* resistant plant species. In recent years, gene expression cassettes have been successfully transferred into many recalcitrant plant species (Guirimand et al., 2009; Liu et al., 2014; Sparks and Jones, 2014; Carqueijeiro et al., 2015; Zhang et al., 2015). The use of bombardment has made it easy to transfer large DNA fragments into the plant genome, though DNA integrity is a concern (Barampuram and Zhang, 2011).

**Figure 3:**
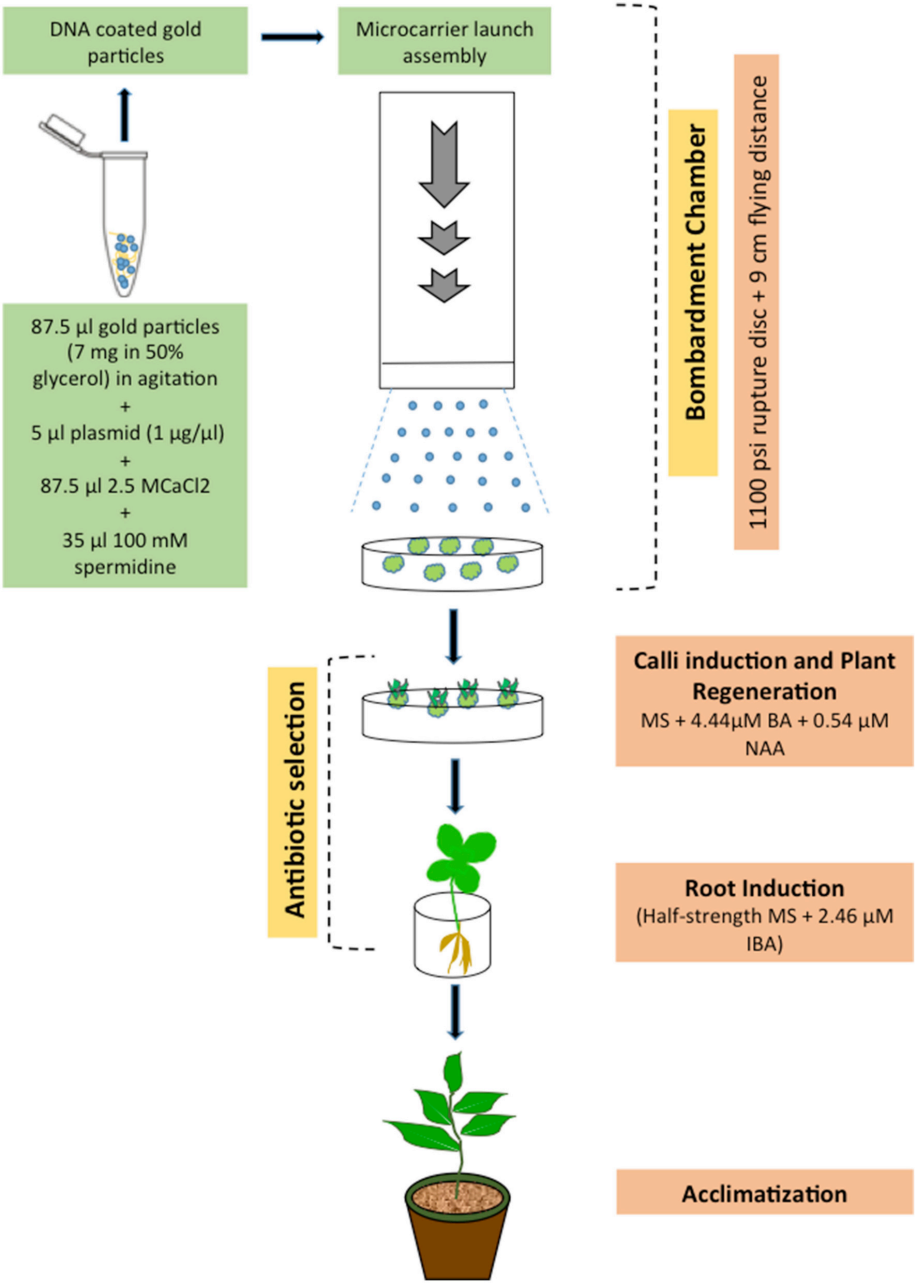
**Scheme showing biolistic bombardment-mediated transformation of ***H. perforatum*** based on the results reported previously (Franklin et al., 2007, 2009b)**.

With the biolistic technology, DNA-coated microparticles (gold or platinum) are accelerated directly into intact tissues by a physical process, thus avoiding the negative influence of *A. tumefaciens* components (elicitors), and genes can be delivered literally into any cell type. Upon reaching the nucleus, the DNA may be integrated, randomly, into the host genome. Since *H. perforatum* remains highly recalcitrant to *A. tumefaciens*-mediated genetic transformation (Franklin et al., 2007, 2008), we have used biolistic bombardment to transform this species (Figure 3). In this work, organogenic nodule explants obtained from cell suspension culture were used as the target materials. The PDS-1000/He particle delivery system (Bio-Rad) was employed to introduce the HPT and GUS genes from the binary vector pCAMBIA1301 into *H. perforatum* tissue. After the selection of bombarded explants, hygromycin-resistant transgenic callus cultures and subsequently GUS positive plants were obtained. Molecular biology methods such as PCR and Southern blot analysis were used to analyze the transgenic nature of resulting plants. The results demonstrated for the first time that *H. perforatum* could be transformed and transgenic plants could be produced *via* biolistic bombardment of novel organogenic cell suspension cultures.

Genotype, physiological age, type of explant, culture period prior to and after gene transfer, culture medium composition, and osmotic pre-treatment were the key parameters affecting efficiency of particle bombardment-mediated transformation. Concerning the biolistic device, the acceleration pressure, the distance between rupture disc, macrocarrier, stopping screen, and target plate, the vacuum pressure in the bombardment chamber, number of bombardments as well as size and density of micro-particles, DNA-micro-particle mixing protocols, and physical configuration of transforming DNA all affected transformation efficiency.

## Future perspectives and strategies for *H. perforatum* transformation

Improving the content of existing bioactive compounds (hypericin, hyperforin, xanthones, etc.) and the production of novel variants are the major targets of *H. perforatum* genetic engineering. Although overexpression of genes involved in the rate limiting biosynthetic steps would allow us to achieve the above goals, pathway engineering in this species is still in its infancy mainly due to the lack of genetic information about these biosynthetic pathways and due to the absence of an efficient transformation method. For instance, although hypericin was identified centuries ago, its biosynthetic pathway is not yet understood. Studies on the genes involved in hypericin biosynthesis have begun only recently and a systematic analysis of genes involved in hypericin biosynthesis has not yet been reported. A decade ago, hypericin biosynthesis was presumed to occur through the polyketide pathway in which type-III polyketide synthases act as key enzymes (Bais et al., 2003). Although a gene termed hyp1 was cloned from red suspension cells and claimed to be involved in the final steps of hypericin biosynthesis (Bais et al., 2003) recent studies contradict its involvement (Karppinen et al., 2008, 2010; Kosuth et al., 2011). The expression pattern of this gene does not correlate with hypericin production, as this gene is constitutively expressed in tissues (roots) and *Hypericum* species that do not produce hypericin (Kosuth et al., 2007). A recent study reported that hyp1 expression is not a limiting factor of hypericin biosynthesis in species that generally produce hypericin (Kosuth et al., 2011). Recently, *de novo* sequencing of *H. perforatum* transcriptomes generated a huge amount of genic data (He et al., 2012; Galla et al., 2015; Soták et al., 2016). In addition, taking advantage of the strong correlation between the presence of dark glands and hypericin accumulation, we performed subtraction between cDNAs of tissues with and without hypericin glands to construct a hypericin gland-specific cDNA library (Singh et al., 2016).

Generally, gene functions can be predicted *via* both forward and reverse genetic approaches. *H. perforatum* possess a polyploid (tetraploid or hexaploid) genome in which genes are usually represented by two or three homoeologous copies with high sequence similarity. Since the effect of single-gene knockouts can generally be nullified by the functional redundancy of homoeologous genes present in the other genomes, forward genetic approaches such as mutagenesis would be inefficient. In plants with polyploid genomes, RNA interference (RNAi) is a valuable technique in which multiple homoeologs can be simultaneously down regulated. RNAi is a double-stranded RNA (dsRNA) induced gene-silencing phenomenon, conserved among various organisms, including animals and plants. RNAi technology has potential to block the activity of enzymes that are not only encoded by a multigene family but are also expressed across a number of tissues and developmental stages. This technology has been successfully used in the dissection of secondary metabolic pathways (Lin et al., 2015). Alternatively, short palindromic repeat (CRISPR)/CRISPR-associated protein (Cas9) system can be used to knockdown gene function (Xing et al., 2014). This system employs an RNA-guided nuclease, Cas9, to induce double-strand breaks. The Cas9-mediated breaks are repaired by cellular DNA repair mechanisms and mediate gene/genome modifications. Although employing the above techniques in *H. perforatum* would be useful to understand gene functions, the RNAi and CRISPR-Cas cassettes need to be introduced into *H. perforatum* genome, which necessitates a robust genetic transformation method. Another powerful reverse genetics approach, which combines chemical mutagenesis with a high-throughput screen for mutations, known as TILLING (Targeting Induced Local Lesions in Genomes) does not require genetic transformation. Although polyploids are well-suited for TILLING due to their tolerance to high mutation densities, this approach is time consuming, laborious and complicated in *H. perforatum* as seed formation in this species proved to be highly polymorphic (Matzk et al., 2001).

Although heterologous expression of secondary metabolic pathway genes has led to the successful production of many secondary metabolites in microbial systems, heterologous expression of *Hypericum*-specific pathways (e.g., hypericin biosynthesis) is currently limited by the lack of cloned genes encoding enzymes involved in the pathways of interest. Moreover, due to the potential toxicity of these compounds to plant tissues, they are accumulated in specialized dark glands. Hence, analyzing the functions of genes related to hypericin synthesis will only be possible in a system, which contain these glands.

Because of the above reasons, establishing an efficient *A. tumefaciens* mediated transformation protocol is unavoidable in order to promote *H. perforatum* functional genomics and metabolic engineering.

Activation of plant defense is considered as a prevailing cause of plant recalcitrance against *Agrobacterium* infection (Franklin et al., 2008; Pitzschke, 2013). Hence, to achieve optimum gene delivery into *H. perforatum* cells via *Agrobacterium*, either suppressing or avoiding the elicitation of defense responses is essential.

*A. tumefaciens*–*H. perforatum* interaction results in the production of ROS. The consequences of the oxidative burst in plant defense responses could be suppressed by the addition of antioxidants such as ascorbic acid, cysteine, citric acid, polyvinylpolypyrrolidone (PVPP), polyvinylpyrrolidone (PVP), dithiothreitol (DTT), BHT, tocopherol, etc. However, it should be recalled that use of these compounds individually did not help in *H. perforatum* transformation in our previous attempts (Franklin et al., 2007). Nevertheless, application of a mixture of antioxidants could be useful, as it has been shown to improve the efficiency of *Agrobacterium*-mediated transformation in a number of other recalcitrant plant species (Dan, 2008; Dan et al., 2010, 2015). It is also important to understand the signaling events that trigger *H. perforatum* defense activation upon *Agrobacterium* interaction. Plant signaling pathways related to systemic resistance and secondary metabolism are involved in the successful activation of defense responses against *A. tumefaciens* (Yuan et al., 2007; Franklin et al., 2008). It should be noted that the involvement of salicylic acid (SA) in shutting down the expression of *A. tumefaciens vir* regulon and thereby directly impairing the infection process has been demonstrated (Yuan et al., 2007; Anand et al., 2008). Therefore, inhibiting the signaling pathways such as SA, methyl jasmonate (MeJ), jasmonic acid (JA), nitric oxide (NO), and the phenylpropanoid pathway using inhibitors [paclobutrazol,2-4-carboxyphenyl-4,4,5,5-tetramethylimidazoline-1-oxyl-3-oxide (cPTIO), 2-aminoindan-2-phosphonic acid (AIP), or diethydithiocarbamate] would be able to improve the transformation rate.

In addition to the above plant defense suppressing strategies, plant defense response against *A. tumefaciens* could be bypassed by applying the following principles. Although *H. perforatum* remains recalcitrant to *Agrobacterium* mediated transformation (Franklin et al., 2007, 2008), we could successfully transform this plant and obtain transgenic plants via particle-bombardment-mediated transformation (Franklin et al., 2007, 2009b) suggesting that *H. perforatum* recalcitrance toward *A. tumefaciens* mediated transformation is conferred by the bacterial components. In spite of the presence of well-characterized pathogen-associated molecular patterns (PAMPs) in *A. tumefaciens* such as flagellin and EF-Tu, it is also known that not all plants respond to all elicitors (Felix and Boller, 2003; Kunze et al., 2004). Hence, it is crucial to identify the specific *A. tumefaciens* elicitors/PAMPs that are recognized by *H. perforatum* to activate its defense machinery, since *A. tumefaciens* devoid of elicitor function would be able to transform *H. perforatum* efficiently. However, it may not be possible to obtain elicitor mutants, if this mutation is lethal.

The plant cell wall plays a crucial role in sensing signals (e.g., wall associated receptor kinases), establishing basal host defense, and serves as a major site of defense activation (Yeom et al., 2012). Presence of plant cell walls can be avoided by using protoplast transformation. Efficient isolation of viable protoplasts from *H. perforatum* and subsequent regeneration is possible (Pan et al., 2004). Taking advantage of the intimate lateral contact of *A. tumefaciens* with plant protoplasts *via* multiple virulent type IV secretion systems (Aguilar et al., 2011), *A. tumefaciens*-mediated T-DNA transfer can be performed (Wang et al., 2005). However, it is possible that protoplasts can produce ROS and soluble phenolics during the maceration process and in response to *A. tumefaciens*. Hence, the chemical inhibitors of the defense pathways and ROS scavengers can be used here, if required. Therefore, it may be possible to transform isolated protoplasts with *A. tumefaciens* at high efficiency. In addition, making use of the fluid-mosaic characteristics of protoplasts, naked DNA uptake methods such as polyethylene glycol (PEG) transfection and electroporation can be achieved (Hassanein et al., 2009), where both the plant cell walls as well as bacterial components are excluded.

Besides the above strategies, virus mediated transformation may be also employed. Viruses infecting *H. perforatum* (Kegler et al., 1999) and *H. japonicum* (Du et al., 2013) have been identified and characterized. Furthermore, nanoparticle mediated DNA delivery into plant cells is gaining momentum (Rai et al., 2015), which would also offer potential benefits in the genetic transformation of *H. perforatum* in the future.

Progress in the areas of *H. perforatum–Agrobacterium* interaction such as understanding the molecular mechanisms of *Agrobacterium* recognition and defense activation together with the novel strategies discussed here will allow us fully exploit and maximize the potential of this tremendously useful source of biotherapeutics.

## Author contributions

GF conceived the idea of this review, designed the overall concept, and participated in the writing. WH and PS participated in the writing of the article and design tables and figures. All the authors approved the final version.

### Conflict of interest statement

The authors declare that the research was conducted in the absence of any commercial or financial relationships that could be construed as a potential conflict of interest.
